# Low Lymphocyte-to-Monocyte Ratio Is the Potential Indicator of Worse Overall Survival in Patients with Renal Cell Carcinoma and Venous Tumor Thrombus

**DOI:** 10.3390/diagnostics11112159

**Published:** 2021-11-21

**Authors:** Łukasz Zapała, Michał Kunc, Sumit Sharma, Wojciech Biernat, Piotr Radziszewski

**Affiliations:** 1 Clinic of General, Oncological and Functional Urology, Medical University of Warsaw, Lindleya 4, 02-005 Warsaw, Poland; sumit.sharma29.06.91@gmail.com (S.S.); pradziszewski@wum.edu.pl (P.R.); 2 Department of Pathomorphology, Medical University of Gdansk, 80-214 Gdańsk, Poland; biernat@gumed.edu.pl

**Keywords:** renal tumor, tumor thrombus, radical nephrectomy, prognostic factors

## Abstract

The purpose of the study was to determine the influence of lymphocyte-to-monocyte ratio (LMR), platelet-to-lymphocyte ratio (PLR), and neutrophil-to-lymphocyte ratio (NLR) values on the prognosis in patients with renal cell carcinoma (RCC) and venous tumor thrombus. The respective data of 91 patients treated with radical surgery in the years 2012–2021 in 2 tertiary referral urological centers were retrieved from local medical databases. Mean calculated 3-year overall survival (OS) reached 70% (mean follow-up 35.3 months). The association between lower LMR and the presence of tumor necrosis (*p* = 0.0004) was observed. Amongst systemic inflammatory markers, only LMR was selected as the sensitive marker predicting death with a calculated cut-off value of 2.53. OS was decreased in patients presenting with low LMR when compared to the high LMR group (39% vs. 82%, *p* = 0.0011). Neither NLR nor PLR were associated with survival rates. In multivariate analysis, LMR was identified as the independent prognostic factor (HR = 0.20, 95% CI 0.07–0.55, *p* = 0.001). Low values of LMR (<2.53) are independently connected with poorer OS in patients with RCC and coexisting tumor thrombus. The incorporation of the hematological variables into the prognostic model greatly increased its accuracy in predicting survival in the distinctive subpopulation of patients with RCC.

## 1. Introduction

There is a growing body of evidence that the connection between renal cancer development and inflammatory processes exists [1]. Several inflammatory markers have prognostic impact in renal cell carcinoma (RCC), including acute phase proteins, e.g., C-reactive protein and ferritin, but also derivatives of complete blood count such as monocytes, platelets, or lymphocytes levels [1,2]. The latter may be combined into lymphocyte-to-monocyte ratio (LMR), platelet-to-lymphocyte ratio (PLR), and neutrophil-to-lymphocyte ratio (NLR). Currently, these are widely recognized as inexpensive systemic inflammatory markers, which are easily calculated from peripheral blood counts [3,4,5]. These laboratory parameters are routinely measured and, therefore, may represent an available tool to assess patient prognosis. Moreover, they might be retrieved from medical records and analyzed retrospectively.

The alterations in NLR, PLR, or LMR in cancer reflect the complex associations between the local immune response with systemic inflammation in various types of cancers [6]. The mechanisms include the accumulation of proinflammatory cytokines within the tumor microenvironment, which may have a direct effect on hematological components, including peripheral blood neutrophil and lymphocyte counts. Moreover, the cytokines may halt the host immune response, inducing the mechanisms of immune evasion in cancer [7]. As a consequence, certain shifts in blood count may indicate the condition of tumor immune microenvironment, e.g., increased numbers of monocytes are thought to represent a high tumor burden [8]. Likewise, elevated neutrophil counts are indicative of tumor progression, and a high NLR has been established as a marker of poor prognosis in multiple human malignancies [9]. In cancer patients a drop in lymphocyte count can reduce anti-cancer immunity [9], while the elevated levels of lymphocytes are associated with a favorable prognosis [10].

RCC is claimed to be a highly immunogenic neoplasm, frequently responsive to immunotherapy, which is currently the mainframe of systemic therapy in metastatic RCC [11]. Many papers to date have focused on the prognostic role of the preoperative inflammatory parameters in the metastatic setting [2,12,13], and only some included localized cases [14]. However, it should be determined whether there is a place for the application of the systemic inflammation markers in the specific subgroup of RCC patients, i.e., with renal tumor venal thrombus. RCC complicated with tumor thrombus in renal vein and inferior vena cava represents a sophisticated clinical scenario, taking into consideration the surgical aspects and poorer prognosis, as claimed by various authors, when compared to tumors limited to the kidney [8,15]. Moreover, the thrombus maintains constant interactions with blood cells and may be the source of substances altering the levels of blood elements [16].

Establishing predictive factors for survival in patients with locally advanced renal cell carcinoma is difficult due to multifactorial issues involved, though. Unfortunately, the clinical course cannot be estimated based only on TNM or Mayo staging, grading, or other pathological features [4,17], and clearly there is a need to determine other reliable prognostic factors. Therefore, the purpose of the study was to determine the influence of MLR, PLR, and NLR values on the prognosis of subpopulations of patients with RCC and tumor thrombus, when hypothesized that changes in the respective inflammatory markers may predict worse outcome.

## 2. Materials and Methods

### 2.1. Study Group

Ninety-one patients with pathologically confirmed RCC and venous thrombus treated with nephrectomy with/without cavotomy and thrombectomy in the period of 2012–2021 in two tertiary referral urological centers were retrieved from local medical databases. Nephrectomy with/without cavotomy and thrombectomy was performed in a systematized manner via lumbotomy or celiotomy procedure. The following data were collected: patients’ age, gender, tumor staging based on CT or MRI scans of chest, abdomen and pelvis according to 2017 TNM classification system [18]; tumor staging using the classification of tumor thrombus level in the Mayo staging system [19,20]; pathological examination report including grade (according to Fuhrman and/or WHO/ISUP when adequate), and presence of necrosis within the tumor; preoperative hematological data (number of neutrophils, platelets, lymphocytes, monocytes along with respective ratios) retrieved from the local certified laboratories (FACS, Sysmex XM200, Sysmex Poland, Poland), as well as the dates of diagnosis and death, and the last follow-up. We excluded any cases with existing inflammatory diseases (i.e., chronic inflammatory conditions, other malignancies, immunosuppression, or autoimmune disease) that would impose possible shifts in the circulating leukocytes. All the patients enrolled had no additional treatments before radical nephrectomy. Overall survival (OS) was determined as the time from the nephrectomy to death from any cause. Finally, telemedicine visits were performed as far as follow-up details were concerned.

### 2.2. Statistical Analysis

The associations between clinicopathological characteristics and morphological parameters were assessed by the U Mann-Whitney test for continuous variables. Categorical variables were compared using chi-square or Fisher’s exact test if applicable. A *p*-value < 0.05 was considered as significant; in cases of multiple comparisons *p*-values were adjusted at a false discovery rate (FDR) = 0.05 using Benjamini-Hochberg correction. Receiver operating characteristic (ROC) curves were plotted for NLR, LMR, and PLR vs. event (death). The optimal cut-off values for each parameter were selected based on the maximal Youden’s index. Differences in OS between groups were assessed using the log-rank test and visualized with Kaplan–Meier curves. Additionally, multivariate Cox proportional hazard analysis with backward selection was performed to create a multivariable model predicting death and to eliminate nonsignificant variables at *p* < 0.05. Statistical analysis was performed with Statistica 13.3 software (TIBCO Software Inc., Palo Alto, CA, USA) and R statistical environment [21]. Boxplots were plotted using the “ggplot2” package Kaplan–Meier curves were plotted using the “survminer” and “ggsci” packages [22,23].

The study was performed under the local ethics committee vote AKBE/72/2021 of the Medical University of Warsaw. Informed consent was obtained from all the subjects involved in the study.

## 3. Results

### 3.1. Baseline Characteristics

The baseline characteristics of the study group are shown in Table 1. The majority of patients were males (54.5%), and the median age of patients was 66 years. Predominantly, patients presented with clear cell carcinoma. The median tumor size was 85.0 mm (interquartile range, IQR—60–110 mm). The median length of hospitalization (LOH) was 8 days (IQR—6–12 days). Most patients developed only minor complications (0–1 according to Clavien Dindo). Adjuvant targeted therapy was administered in 20 (22%) cases (tyrosine kinase inhibitors—18, monoclonal antibodies—2).

### 3.2. Survival Analysis

Mean calculated 3-year OS reached 70%, with mean follow-up 35.3 months (median 27, range 1–109).

### 3.3. Univariate and Multivariate Analysis

**Figure 1:**
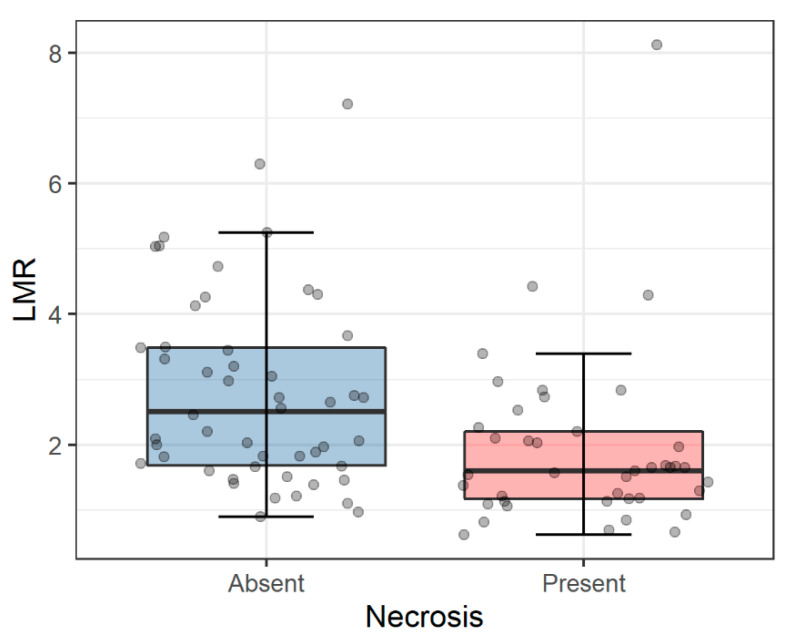
Associations between LMR and tumor necrosis. The presence of necrosis was associated with significantly lower LMR (*p* = 0.0004, Mann-Whitney U test). Horizontal lines inside boxes show median values. The lower and upper hinges correspond to the first and third quartiles (the 25th and 75th percentiles). Upper and lower whiskers indicate 1.5 interquartile range of the lower and upper quartile, respectively. Circles represent individual measures. Abbreviations: LMR: lymphocyte-to-monocyte ratio; PLR: platelet-to-lymphocyte.

Associations between morphological parameters and selected clinicopathological variables are shown in Table 2. After correction for multiple comparisons, we found the association between lower LMR and tumor necrosis (*p* = 0.0004, q = 0.007) as presented in Figure 1.

Additionally, there was a trend toward lower LMR values in males than in females (*p* = 0.008, q = 0.07) (Table 2). No associations were found between NLR, PLR, and LMR and nodal status, presence of distant metastases, tumor grade, and Mayo stage, respectively.

**Figure 2:**
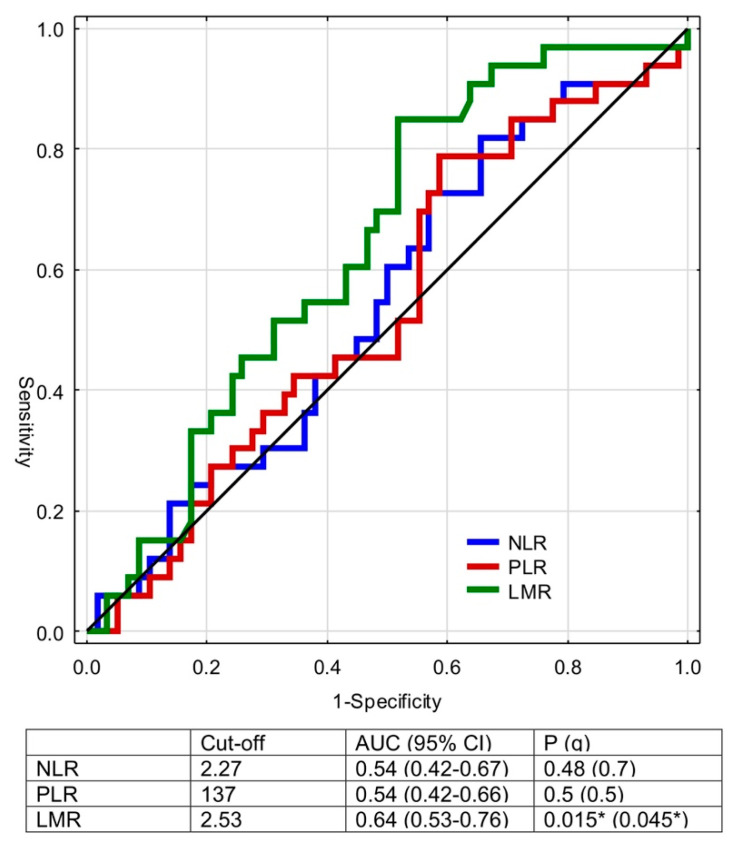
Receiver operating curves for NLR, LMR, PLR, versus event (death). Q values were calculated with Benjamini-Hochberg correction. *—statistically significant. Abbreviations: NLR: neutrophil-to-lymphocyte ratio; LMR: lymphocyte-to-monocyte ratio; PLR: platelet-to-lymphocyte.

The calculated cut-off values for NLR, LMR, and PLR to predict death were 2.27 (*p* = 0.48), 2.53 (*p* = 0.015), and 137 (*p* = 0.5), respectively (Figure 2). Figure 2 demonstrates ROC and areas under the curve.

**Figure 3:**
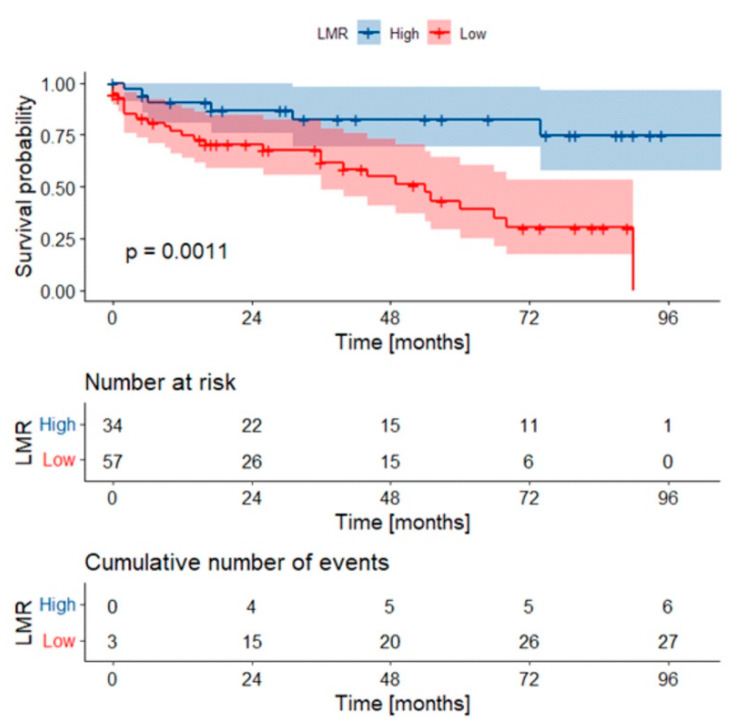
Kaplan-Meier curves with confidence intervals for overall survival stratified by LMR values. *p*-value was calculated with log-rank test. Abbreviations: LMR: lymphocyte-to-monocyte ratio. Low LMR: below cut-off.

When analyzing the preoperative systemic inflammatory markers, the 5-year OS rate was lower in patients presenting with low LMR (below the cut-off value of 2.53), when compared to high LMR values (39% vs. 82%, *p* = 0.0011) (Figure 3). Neither NLR nor PLR was significantly associated with survival rate.

The following variables were taken into consideration during the generation of the multivariable Cox regression model: nodal status, the presence of distant metastases, tumor grade, tumor necrosis, length of hospitalization, Clavien-Dindo grade, gender, and Mayo stage. The backward selection was performed to create the multivariable model predicting death in the cohort. Finally, regional lymph node status (*p* < 0.001), tumor grade (*p* = 0.004), and LMR values (*p* = 0.001) were incorporated into the model (Table 3).

## 4. Discussion

In the current paper, we focused on the assessment of the role of hematological markers in the survival prognosis of patients with RCC and tumor thrombus. Many studies have investigated the relationship between the blood parameters and prognosis in kidney cancer [1,24]. Unveiling risk factors connected with postoperative mortality is critical due to the coexisting surgical complexity in the case of tumor thrombus [25]. Some authors speculate that RCC with tumor thrombus may promote cytokine release and systemic inflammation [16]. There is a lack of papers regarding NLR, PLR, and LMR application in the pretreatment prognostic models to stratify the RCC cases with venous involvement. Here, we showed that higher preoperative LMR was independently correlated with better OS for patients with RCC and tumor thrombus.

Firstly, we aimed at finding any correlations between morphological parameters and selected clinicopathological variables. Interestingly, no associations were recognized concerning NLR, PLR, and LMR and nodal status, presence of distant metastases, tumor grade, and Mayo stage, respectively. It seems that these markers might be independent of tumor burden in RCC with venous thrombus. Otunctemur et al. described that patient with a higher grade and stage have elevated levels of NLR based on the observations of the cohort of 432 cases with RCC staged T1–4 who underwent radical or partial nephrectomy [26]. However, Arda et al. in the population of nonmetastatic RCC (T1–4N0M0) cases did not find associations between grade and lymphocyte, neutrophil, and platelet counts, and NLR values [1]. Thus, it is important to further stratify the subgroups of patients with RCC with special interest to cases with venous involvement as no such data exist in the literature.

Here, we noticed a trend towards the association between elevated LMR values and male gender. Hutterer et al. described the significant relationship between low LMR and older age (≥65 year), high tumor grade (G3), and advanced pathologic T descriptor (pT3) [27]. Furthermore, similarly to our observations, the presence of histologic tumor necrosis and male gender were associated with lower LMR, as well [27]. In the paper by Rajwa et al. [14], low LMR was found in cases with higher stages and in the presence of tumor necrosis.

Additionally, the authors revealed similar findings in the case of both high levels of PLR and NLR [14]. However, we did not find a correlation in our study. One of possible explanations is that the prognostic value of PLR in RCC remains inconsistent, on the contrary to the observations received from studies on other cancers [28]. During tumorigenesis, inflammatory mediators promote the recruitment of megakaryocytes causing thrombocytosis [6] that one would connect with the formation of the tumor thrombus. Corresponding findings were described in the paper by Hu et al., in which elevated PLR was found to be associated with high tumor stage in Mayo scale but, interestingly, not the depth of invasion when subgrouped into T1–2 and T3–4 in TNM or nodal status [29].

Then, we focused on the estimation of the cut-off values of NLR, PLR, and LMR in prognosis analysis. Here, only the LMR showed the significant area under the curve, and the estimated cut-off value for this parameter reached 2.53. The diversification of patients according to this threshold was identified as an independent prognostic factor for OS as presented in Figure 2. On the one hand, monocytes transform into tumor-associated macrophages with pro-cancerous properties, i.e., promotion of neoangiogenesis and tumor cell growth, migration, and metastases [6]. On the other, decreased lymphocyte count may result in the attenuation of immunologic antitumor reaction and peripheral lymphopenia is a marker of poor prognosis in RCC patients [30]. Due to the imbalance caused by tumor-associated factors, the induction of the effector cells, such as lymphocytes or monocytes, is disturbed [6]. LMR may therefore mirror the condition of antitumor immunity and help to estimate the prognosis of patients with RCC and tumor thrombus. In the current paper, we have presented that low LMR was associated with the significantly worse OS, but no such relationship was revealed, as far as NLR and PLR were concerned. In the large Austrian study by Hutterer et al. on 687 individuals, LMR < 3 was indicative of 2.3-fold increase in the risk of death due to RCC [27]. It was confirmed by Chinese authors in the retrospective analysis of 430 RCC cases staged T1–3N0M0, in which LMR was found to increase the accuracy of the existing prognostic models in case of intermediate and high-risk patients [5].

Recent PubMed database review by Boissier et al. concluded that generally NLR < 3 could be a discriminative value for prediction of survival rather metastatic than localized RCC [17]. In our setting, based on internal cut point analysis a preoperative threshold of 2.27 in the case of NLR was implemented followed by an insignificant effect of the cut-off used on the prognosis. Prior publications assessing NLR have not exclusively examined RCC patients with tumor thrombus [14,25]. One of the possible explanations comes from the paper by Peyton et al. [25]: NLR > 4.0 enabled the researchers to discriminate patients with significantly shorter survival based on 332 metastatic cases with coexisting tumor thrombus. In our cohort 97% of patients presented with T3a disease, which may result in similar but not the same results to localized cases (stages pT1–3) from the literature [17].

As far as cut-off values are concerned, in the paper by Hu et al. [29], the threshold for PLR determined in ROC curves analysis was 185 and occurred to correlate with the worse OS if elevated. When subgrouping into the region of publication, therapeutic intervention, and sample size to overcome the heterogeneity of the RCC cases, Wang et al. revealed that PLR predicted worse OS in Asian patients, metastatic cases receiving mixed therapies, and targeted therapies and in cohorts >100 cases [28]. Taking into consideration the relatively low frequency of tumor thrombus cases, one should be aware of the last factor as well.

Finally, the multivariable model predicting death in patients with RCC and tumor thrombus was developed. We found that the addition of one of the inflammatory markers improved the discriminatory performance and the model finally included regional lymph node status, tumor grade, and LMR values. There is a general tendency in the literature to incorporate some of the hematological parameters in the predictive tools, especially in the metastatic setting [11]. Here we present for the first time the usefulness of LMR in the subpopulation of RCC cases with tumor thrombus. In general, the inclusion of inflammatory markers into the prognostic models increases their accuracy but not to the extent that histopathological variables would be omittable [7,25].

The limitations of this paper are mainly related to its retrospective design and, therefore, obvious biases do exist. Data on OS only were available in this database as opposed to the determination of death due to RCC. The model presented above definitely needs external validation, even though the data come from two tertiary centers. Presumably, though, the study raises for the first time the utility of hematological parameters in the RCC with tumor thrombus in a relatively large cohort with potentially useful clinical implications.

## 5. Conclusions

In conclusion, high LMR (>2.53) was independently associated with better OS in patients with RCC and coexisting tumor thrombus. The incorporation of the hematological variables into the prognostic model greatly increased its accuracy in predicting survival in this high-risk subpopulation of individuals with RCC. This sheds some light on the inflammatory mechanism involved in the natural history of RCC and enables further stratification of the patients into the respective subgroups for follow-up purposes and, possibly, additional systemic treatment in the future.

## Figures and Tables

**Table 1:** Basic characteristics of the study group. Abbreviations: LMR: lymphocyte to monocyte ratio; NLR: neutrophil to lymphocyte ratio; PLR: platelet to lymphocyte ratio; IQR interquartile range.

Characteristic		N (%)
Gender	Male	50 (54.5)
	Female	41 (45.5)
Age	Median: 66 (IQR 60–71 years)	
Histology	Clear cell	88 (97)
	other	3 (3)
Grade	Low (G1–2)	28 (30)
	High (G3–4)	63 (70)
T	3a	88 (97)
	3b	1 (1)
	3c	-
	4	2 (2)
N	0	78 (86)
	1	13 (14)
M	0	67 (74)
	1	24 (26)
R	0	70 (77)
	1–2	21 (23)
Tumor necrosis	Present	41 (45.5)
	absent	50 (54.5)
Mayo stage	Low (0–1)	85 (93)
	High (≥2)	6 (7)
LOH	Short (≤9 days)	61 (67)
	Long	29 (33)
Clavien-Dindo	Low (0–1)	79 (87)
	High ≥2	12 (13)
Death during follow-up	No	58 (64)
	Yes	33 (36)
Preoperative values	Lymphocytes	median 1.55 IQR—1.18–2.12
	Monocytes	median 0.82 IQR—0.6–1.04
	Neutrophils	median 4.91 IQR—3.8–6.26
	Platelets	median 262.5 IQR—220–334
	LMR	median—1.96 IQR—1.41–3.05
	NLR	median—3.18 IQR—2.04–4.79
	PLR	median—166 IQR—119–234

**Table 2:** Associations between morphological parameters and clinicopathological variables. Given values: median (IQR). *p* values were calculated with U Mann-Whitney test. q values were calculated with Benjamini-Hochberg correction. *—statistically significant. Abbreviations: LMR: lymphocyte to monocyte ratio; NLR: neutrophil to lymphocyte ratio; PLR: platelet to lymphocyte ratio.

Feature	NLR	*p* (q)	PLR	*p* (q)	LMR	*p* (q)
Sex						
Male	3.47 (2.32–4.79)	0.16 (0.28)	172 (127–226)	0.8 (0.9)	1.67 (1.26–2.53)	0.008 * (0.07)
Female	2.86 (1.88–4.26)		160 (116–233)		2.64 (1.57–3.49)	
Nodal status						
N0	3.12 (2.04–4.78)	0.86 (0.9)	160 (117–249)	0.66 (0.85)	2.01 (1.46–3.10)	0.15 (0.34)
N1	3.34 (2.30–4.26)		188 (157–229)		1.60 (0.97–2.20)	
Distant metastases						
M0	2.95 (1.90–4.57)	0.15 (0.31)	156 (113–222)	0.021 * (0.126)	2.06 (1.47–3.44)	0.02 * (0.11)
M1	3.63 (2.76–5.21)		217 (151–260)		1.66 (1.18–2.15)	
Grade						
Low (1–2)	3.39 (1.74–4.59)	0.63 (0.88)	155 (104–230)	0.32 (0.47)	1.82 (1.49–2.93)	0.76 (0.9)
High (3–4)	3.07 (2.04–5.16)		178 (120–249)		2.03 (1.39–3.05)	
Necrosis						
No	2.82 (2.00–4.25)	0.03 * (0.11)	163 (116–217)	0.24 (0.39)	2.51 (1.67–3.49)	0.0004 * (0.007) *
Yes	3.73 (2.77–5.56)		181 (128–258)		1.60 (1.17–2.21)	
Mayo stage						
Low	3.18 (2.05–4.74)	0.1 (0.3)	166 (119–249	0.9 (0.9)	2.00 (1.46–3.10)	0.1 (0.26)
High	2.93 (1.67–5.73)		176 (128–226)		1.46 (1.09–2.10)	

**Table 3:** Multivariable model predicting death in the current cohort. *—statistically significant. Abbreviations: LMR—lymphocyte to monocyte ratio.

Feature	HR (95% CI)	*p*
Nodal status (N0 vs. N1)	0.19 (0.07–0.47)	0.0003 *
Grade (High vs. Low)	3.92 (1.56–9.86)	0.004 *
LMR (High vs. low)	0.20 (0.07–0.55)	0.001 *

## Data Availability

The data presented in this study are available on reasonable request from the corresponding authors.
